# Toxicological insights and safety considerations of vorasidenib in grade 2 astrocytoma and oligodendroglioma

**DOI:** 10.1097/JS9.0000000000002356

**Published:** 2025-04-01

**Authors:** Gabriel Vinícius Rolim Silva, M. Aktaruzzaman, Umberto Laino Fulco, Taha Alqahtani, Magdi E A Zaki, Jonas Ivan Nobre Oliveira

**Affiliations:** a Department of Biophysics and Pharmacology, Bioscience Center, Federal University of Rio Grande do Norte, Natal/RN, Brazil; b Department of Pharmacy, Faculty of Biological Science and Technology, Jashore University of Science and Technology, Jashore, Bangladesh; c Department of Pharmacology, College of Pharmacy, King Khalid University, Abha, Saudi Arabia; d Department of Chemistry, College of Science, Imam Mohammad Ibn Saud Islamic University (IMSIU), Riyadh, Saudi Arabia

**Keywords:** vorasidenib, IDH 1/2 inhibition, toxicity

## Abstract

Vorasidenib, a dual inhibitor of isocitrate dehydrogenase 1 and 2 (IDH1/2), has shown promise as a therapeutic agent following its recent FDA approval for the treatment of grade 2 astrocytomas and oligodendrogliomas harboring IDH mutations in patients 12 years of age and older following surgery. While Vorasidenib offers significant potential in targeting altered metabolic pathways in low-grade gliomas, its comprehensive toxicologic and safety profile has not been adequately explored. This research letter addresses this critical gap by presenting an *in silico* analysis of the potential toxicologic effects of Vorasidenib. Using computational tools – ADMETlab 3.0, FAF-Drugs 4.1, DeepPK, vNN-ADMET, Pred-hERG 5.0, ADVERPred, PreADMET, and ADMET-AI – and databases such as ChEMBL, PubChem, and ChemSpider, we evaluated the key physicochemical properties and predicted ADMET profiles of Vorasidenib, along with a comparative analysis of two other drugs, namely Ivosidenib and Enasidenib. Our results suggest potential risks associated with drug-induced liver injury (DILI) and hepatotoxicity, with structural properties indicative of hepatocellular damage during and after treatment. The low clearance rates associated with the low maximum recommended dose suggest that Vorasidenib may accumulate in the bloodstream over time, increasing the likelihood of toxic reactions. In addition, the predictive models indicate concerns for neurotoxicity, nephrotoxicity and cardiotoxicity, including potential blockade of hERG channels leading to QT interval prolongation and cardiac arrhythmias. Importantly, the analysis also indicates risks of genotoxicity and carcinogenicity, raising concerns about promoting additional tumor formation in patients already prone to malignancies. These results emphasize the need for further preclinical and clinical studies to validate the safety of Vorasidenib. A comprehensive understanding of the toxicologic profile is critical to ensure that the therapeutic benefit for patients with IDH1/2-mutated low-grade gliomas is not compromised by potential adverse effects. Careful monitoring of patients and tailored therapeutic strategies are essential to optimize clinical outcomes and guide physicians in the safe use of Vorasidenib in clinical practice.

**HIGHLIGHTS**

- Vorasidenib, an IDH1/2 dual inhibitor, holds therapeutic promise for grade 2 astrocytoma and oligodendroglioma.
- *In silico* toxicologic analysis indicates risks of hepatotoxicity, neurotoxicity, nephrotoxicity, and cardiotoxicity with Vorasidenib use.
- Low clearance rates suggest possible Vorasidenib accumulation, raising risk of prolonged toxic reactions in patients.
- Predictive models highlight genotoxic and carcinogenic concerns, especially in patients already predisposed to malignancies.
- Further preclinical and clinical studies are needed to validate the safety profile of Vorasidenib.

## Background

The management of low-grade gliomas (LGGs), such as Grade 2 astrocytomas and oligodendrogliomas, presents a unique set of challenges that are often overshadowed by the more aggressive high-grade gliomas. These tumors arise from glial cells are characterized by slow growth that often remains asymptomatic for long periods of time^[^1^]^. Each year, approximately 0.5 to 1.0 per 100 000 individuals are diagnosed with an LGG, accounting for about 3500 new cases annually in the United States alone^[^2^]^.

While LGGs are classified primarily as World Health Organization (WHO) Grades II and III and are distinguished by their relatively indolent nature compared to high-grade gliomas, they are far from benign. A substantial proportion of patients experience malignant transformation, significantly worsening prognoses and necessitating more aggressive interventions^[^3,4^]^. Additionally, recurrence rates for LGGs are high, with many cases eventually progressing to high-grade gliomas, complicating long-term management^[^4,5^]^. Despite being labeled as low grade, these tumors pose a significant long-term risk, with 5-year survival rates of 51.6% for astrocytomas and 82.7% for oligodendrogliomas, and median survival times ranging from 5 to 8 years and 6–7 years, respectively^[^6^]^.

Beyond survival statistics, the burden of LGGs extends to profound cognitive, neurological, and economic dimensions. Neurological symptoms, such as seizures, headaches, and cognitive deficits, are pervasive and can severely diminish patients’ quality of life (QoL). These impacts necessitate of tumor control and comprehensive cognitive rehabilitation to address deficits in memory, executive function, and emotional well-being^[^7^]^. Furthermore, QoL concerns often influence treatment decisions, with many patients prioritizing functionality and independence over survival alone^[^8^]^.

The economic burden of LGGs is equally significant, encompassing direct medical costs and indirect costs associated with reduced productivity and long-term caregiving. Direct healthcare expenditures are driven by frequent imaging, surgical interventions, chemotherapy, and radiotherapy, as well as the management of treatment-related toxicities^[^9,10^]^. In the United States, the cost of glioma treatment can exceed $200 000 per patient annually, with LGGs contributing to a substantial share of this financial burden due to their chronic and recurrent nature^[^8^]^. Indirect costs, including lost wages and caregiver absenteeism, further amplify the economic impact. A study in Europe estimated that the indirect costs of gliomas surpass €400 000 per premature death, underscoring the societal implications of this disease^[^8^]^.

Astrocytomas and oligodendrogliomas exhibit a complex molecular landscape, prominently characterized by frequent mutations in isocitrate dehydrogenase (IDH) genes and the 1p/19q codeletion, alongside various histopathological parameters^[^11^]^. In lower-grade astrocytomas, IDH mutations are particularly prevalent: mutations in isocitrate dehydrogenase-1 (IDH1) occur in ~80% of Grade 2 and Grade 3 diffuse gliomas, while isocitrate dehydrogenase-2 (IDH2) mutations are observed in about 4% of cases^[^12^]^. These genetic alterations lead to the accumulation of the oncometabolite 2-hydroxyglutarate (2-HG), which contributes to tumorigenesis and disrupts normal cellular functions^[^11^]^.

Currently, Grade 2 astrocytomas or oligodendrogliomas harboring susceptible IDH1 or IDH2 mutations remain incurable, and existing therapies are associated with substantial toxicity^[^13,14^]^. For patients with low-risk Grade 2 tumors, the standard approach typically involves surgical resection followed by observation, primarily due to the toxic side effects linked to available chemotherapeutic agents^[^15^]^. In contrast, high-risk Grade 2, as well as Grades 3 and 4 astrocytomas or oligodendrogliomas, are managed with resection followed by postoperative chemotherapy^[^16^]^.

The initial clinical development of first-in-class mutant IDH inhibitors, such as Ivosidenib and Enasidenib, focused on relapsed or refractory acute myeloid leukemia (AML) harboring IDH1^[^17^]^ or IDH2 mutations^[^18^]^, respectively. Ivosidenib, an IDH1 inhibitor, was the first to advance to clinical trials for glioma treatment^[^19^]^. In this study, Ivosidenib achieved a median progression-free survival (PFS) of 13.6 months (95% CI: 9.2–33.2 months) for patients with nonenhancing gliomas and 1.4 months (95% CI: 1.0–1.9 months) for those with enhancing gliomas. However, the need for therapies specifically designed for central nervous system (CNS) penetration remained.

Building upon these findings, Vorasidenib – a novel dual inhibitor of mutant IDH1 and IDH2 with enhanced CNS penetration – was developed (^[^20,21^]^. In a Phase I clinical trial, Vorasidenib achieved a median progression-free survival (PFS) of 36.8 months in patients with non-enhancing gliomas, compared to 3.6 months in those with enhancing gliomas^[^21^]^. Preclinical studies revealed that Vorasidenib inhibits wild-type and mutant IDH1/IDH2, reduces 2-hydroxyglutarate (2-HG) production, and partially restores cellular differentiation^[^22^]^. Furthermore, Vorasidenib demonstrated effective brain penetration in multiple preclinical models and inhibited 2-HG production by over 97% in glioma tissue in an orthotopic glioma mouse model^[^20^]^. A randomized perioperative study comparing Ivosidenib and Vorasidenib further showed that both agents reduced tumor 2-HG concentrations by over 90%, decreased tumor cell proliferation, and reversed gene expression programs associated with IDH-mutant gliomas^[^23^]^.

A pivotal double-blind Phase III trial (INDIGO; ClinicalTrials.gov number NCT04164901) recently demonstrated that Vorasidenib significantly prolongs PFS and delays the time to next intervention (TTNI) in adults with recurrent or progressive IDH1/2-mutant Grade 2 gliomas^[^24^]^. The median PFS was 27.7 months for patients receiving Vorasidenib, compared to 11.1 months for those on placebo, resulting in a hazard ratio of 0.39 (*P* < 0.001). Furthermore, the time to the next anticancer intervention was significantly extended in the Vorasidenib group, indicating that patients experienced a substantial delay in requiring additional therapy.

Recognizing these promising results, on 6 August 2024, the U.S. Food and Drug Administration (FDA) approved Vorasidenib (VORANIGO or AG-881) for the treatment of Grade 2 astrocytomas or oligodendrogliomas with susceptible IDH1 or IDH2 mutations in patients who have undergone surgery. This approval marks a significant milestone, offering a new therapeutic option for these challenging tumors.

Despite this progress, optimal management strategies for LGGs remain a topic of ongoing discussion. For patients with low-risk LGGs, a watch-and-wait approach following surgery is often adopted to delay the initiation of therapies associated with significant toxicities^[^25^]^. However, this strategy carries the risk of disease progression and malignant transformation during the observation period.

Introducing targeted therapies like Vorasidenib during the active observation phase presents an opportunity to delay the need for more toxic treatments and preserve quality of life, especially in younger patients. However, understanding the long-term safety profile of Vorasidenib is crucial. While clinical trials have demonstrated its efficacy, the potential for adverse reactions and toxicities with prolonged use remains a concern.

Historically, medications have been withdrawn from the market when toxicities not detected during clinical trials emerge after widespread use. For instance, troglitazone, a drug for type 2 diabetes, was removed due to hepatotoxicity that was not fully apparent during initial trials^[^26^]^. Even recently approved medications continue to reveal new adverse effects postmarket, highlighting the importance of ongoing safety monitoring^[^27^]^,

In our study, we utilized in silico tools to predict the toxicological properties and potential adverse reactions of Vorasidenib that may emerge with long-term use. By assessing its Absorption, Distribution, Metabolism, Excretion, and Toxicity (ADMET) profiles, we aim to optimize its therapeutic use and ensure patient safety. This approach is particularly pertinent given that some toxicities may arise due to genetic variations affecting drug metabolism, which can lead to severe adverse reactions^[^28^]^.

The introduction of Vorasidenib raises hopes of improving long-term patient outcomes and reducing the burden on healthcare systems. However, it also underscores the need for comprehensive safety evaluations to anticipate and mitigate potential risks, balancings its projected effectiveness in the treatment of grade 2 gliomas with possible risks of acute and long-term toxicities. Our research contributes to this critical aspect by providing an in-depth analysis of Vorasidenib’s toxicological profile using advanced computational methods, thus offering a broader look into the drug’s pharmacokinetic and toxicity parameters with the goal of facilitating a more individual approach on its usage, such as the monitoring of liver function enzymes during treatment. This not only aids in the safe integration of Vorasidenib into clinical practice but also exemplifies a proactive approach to drug safety in the era of personalized medicine.

## Methods

We conducted a detailed *in silico* analysis to evaluate Vorasidenib’s drug-likeness and toxicity properties using computational platforms and drug databases. The compound’s canonical SMILES (C[C@H](C(F)(F)F)NC1 = NC(=NC(=N1)C2 = NC(=CC = C2)Cl)N[C@H](C)C(F)(F)F) was obtained from **PubChem** (https://pubchem.ncbi.nlm.nih.gov/docs/statistics), a database containing over 100 million unique compounds. Additional data were retrieved from **ChemSpider** (https://www.chemspider.com/) and **ChEMBL** (https://www.ebi.ac.uk/chembl/)—two other databases containing over 120 million structures and 2.5 million compounds, respectively—to analyze its physicochemical properties, including density, flexibility, formal charge, fraction of sp^3^-hybridized carbons (Fsp^3^), hydrogen bond acceptors (HBA), hydrogen bond donors (HBD), total hydrogen bonding capacity (HBA + HBD), Log D, Log P, intrinsic aqueous solubility (log Sw), maximum ring system size, molecular weight (MW), number of carbon atoms (n_carbon), heavy atoms (n_HeavyAtoms), heteroatoms (nHet), ring systems (n_SystemRing), formal charges (NumCharges), hydrogen-to-carbon ratio (ratioH_C), number of rigid bonds (nrb), rotatable bonds (nrotb), solubility, solubility forecast index, stereocenters (StereoCenters), total charge (TotalCharge), topological polar surface area (tPSA), and van der Waals volume (VdW). Vorasidenib was selected through FDA’s own database of newly approved drugs, whereas Ivosidenib and Enasidenib were screened and selected in accordance with their generic names, pharmacological class, and clinical usages and applications, with the goal of selecting suitable control drugs to compare Vorasidenib’s pharmacological profile with, and discarding other antineoplastic drugs that were either used for other purposes, or not as widely utilized as those two.

To comprehensively predict the ADME-Toxicity properties of Vorasidenib, we utilized advanced AI-driven platforms, including **ADMETlab 3.0** (https://admetlab3.scbdd.com/), **FAF-Drugs 4.1** (https://mobyle2.rpbs.univ-paris-diderot.fr/cgi-bin/portal.py#forms::FAF-Drugs4), **Deep-PK** (https://biosig.lab.uq.edu.au/deeppk/), **vNN-ADMET** (https://vnnadmet.bhsai.org/vnnadmet/home.xhtml), **Pred-hERG 5.0** (http://predherg.labmol.com.br/), **pkCSM** (https://biosig.lab.uq.edu.au/pkcsm/prediction) **ADVERPred** (https://www.way2drug.com/adverpred/), **PreADMET** (https://preadmet.webservice.bmdrc.org/) and **ADMET-AI** (https://admet.ai.greenstonebio.com/), utilizing the standard parameters given for each tool, along with the respective SMILES for each drug^[^29-36^]^. These platforms allowed us to assess various toxicity parameters such as:

1. **Organ Toxicity**: Evaluation of hepatotoxicity, immunotoxicity, neurotoxicity, nephrotoxicity, ototoxicity, hemotoxicity, hemolytic toxicity, cytotoxicity, cardiotoxicity (general, hERG I and hERG II inhibition, myocardial infarction, heart failure, arrhythmia), skin sensitivity, acute dermal toxicity, ocular irritation and corrosion, maximum-tolerated dose in humans (Log mg/kg/day), respiratory toxicity, mitochondrial toxicity, and reproductive toxicity.
2. **Genotoxicity**: Assessment of mutagenicity (Ames test), genotoxicity rules (carcinogenicity/mutagenicity), nongenotoxic carcinogenicity, carcinogenicity in mice and rats, and micronucleus analyses.
3. **Ecotoxicity**: Environmental impact assessments, including acute and general aquatic toxicity, toxicity to fish and bees, biodegradability, Ames toxicity, acute oral toxicity in rats, chronic oral toxicity in rats (LOAEL), T. pyriformis toxicity, and toxicity to Salmonella strains.

Toxicity predictions were further validated using additional tools such as VirtualToxLab to ensure safety before experimental validation. Special focus was given to (i) metabolic and endocrine disruption, (ii) cardiotoxicity, and (iii) carcinogenicity using VirtualToxLab. Equally important, (iv) the presence of toxicophores, (v) hepatotoxicity, and (vi) cardiac toxicity via hERG inhibition were analyzed using FAFDrug4, pkCSM server, and Pred-hERG, respectively. This multifaceted approach ensures a robust evaluation of Vorasidenib’s safety profile, supporting its potential therapeutic application while mitigating risks associated with long-term use. Table 1 showcases the available data set and area under curve (AUC) for statistical validation of our toxicity results for some of the main tools used in our research^[^29,33-35^]^.

**Table 1:** Analyzed toxicity, values found for each molecule, tool utilized, clinical significance, and statistical parameters available on that webserver. For toxicity parameters with multiple webservers employed, we selected the ones with highest data set and best AUC values. Parameters marked with (%) indicate probability of that toxicity being present (values ranging from 0.0 to 1.0). AUC values marked as n/a are not probabilistic and thus do not apply.

Toxicity	Vorasidenib	Ivosidenib	Enasidenib	Tool	Data Set	Clinical Significance	AUC
hERG blocker (%)	0.504	0.875	0.516	ADMETlab 3.0	13 845	Cardiac toxicity	0.935
DILI (%)	0.465	0.798	0.515	ADMETlab 3.0	467	Liver toxicity	0.900
Hepatotoxicity (%)	0.813	0.964	0.973	ADMETlab 3.0	2304	Liver toxicity	0.765
AMES toxicity (%)	0.064	0.143	0.08	ADMETlab 3.0	7575	Mutagenicity	0.888
Rat oral acute toxicity (%)	0.293	0.816	0.797	ADMETlab 3.0	7327	Lethal dose (LD_50_)	0.855
Skin sensitization (%)	0.199	0.385	0.379	ADMETlab 3.0	405	Skin irritation	0.764
Carcinogenicity (%)	0.634	0.244	0.294	ADMETlab 3.0	1041	Oncogenesis	0.742
Eye corrosion (%)	0.001	0.000	0.000	ADMETlab 3.0	2298	Ocular lesion	0.988
Eye irritation (%)	0.765	0.007	0.617	ADMETlab 3.0	5219	Ocular lesion	0.981
Respiratory (%)	0.795	0.300	0.997	ADMETlab 3.0	1388	Respiratory system toxicity	0.881
Drug-induced nephrotoxicity (%)	0.963	0.990	0.997	ADMETlab 3.0	565	Nephrotoxicity	0.807
Drug-induced neurotoxicity (%)	0.894	0.918	0.750	ADMETlab 3.0	684	Neurotoxicity	0.820
Ototoxicity (%)	0.768	0.926	0.927	ADMETlab 3.0	2808	Hearing loss	0.790
Hematotoxicity (%)	0.178	0.232	0.225	ADMETlab 3.0	2374	Blood cells toxicity	0.794
Genotoxicity (%)	0.985	1.000	1.000	ADMETlab 3.0	641	Oncogenesis	0.953
RPMI-8226 immunotoxicity (%)	0.113	0.114	0.132	ADMETlab 3.0	48 811	White blood cells toxicity	0.888
A549 cytotoxicity (%)	0.393	0.239	0.839	ADMETlab 3.0	3302	Lung oncogenesis	0.853
Hek293 cytotoxicity (%)	0.497	0.575	0.973	ADMETlab 3.0	6898	Kidney oncogenesis	0.866
BCF (log)	2.290	1.238	1.529	ADMETlab 3.0	676	Bioaccumulation	n/a
IGC50 (log)	3.690	3.750	3.450	ADMETlab 3.0	1787	Environmental toxicity	n/a
LC50DM (log)	5.130	5.440	4.610	ADMETlab 3.0	816	Acute toxicity	n/a
LC50FM (log)	4.560	4.760	4.420	ADMETlab 3.0	347	Aquatic environment toxicity	n/a
NR-AhR (%)	0.7–1.0	0–0.3	0.7–1.0	ADMETlab 3.0	6603	Oncogenesis	0.930
NR-AR (%)	0–0.3	0.3–0.7	0–0.3	ADMETlab 3.0	7312	Endocrine system toxicity	0.893
NR-Aromatose (%)	0–0.3	0.3–0.7	0.7–1.0	ADMETlab 3.0	5887	Endocrine system toxicity	0.904
NR-ER (%)	0–0.3	0–0.3	0.7–1.0	ADMETlab 3.0	6166	Endocrine system toxicity	0.817
NR-ER-LBD (%)	0–0.3	0–0.3	0–0.3	ADMETlab 3.0	7052	Endocrine system toxicity	0.894
NR-PPAR-gamma (%)	0.3–0.7	0–0.3	0–0.3	ADMETlab 3.0	6586	Endocrine system toxicity	0.907
SR-ARE (%)	0.7–1.0	0–0.3	0.7–1.0	ADMETlab 3.0	5652	Metabolism inhibition	0.870
SR-ATAD5 (%)	0–0.3	0–0.3	0.3–0.7	ADMETlab 3.0	7170	Oxidative stress	0.929
SR-HSE (%)	0.7–1.0	0–0.3	0–0.3	ADMETlab 3.0	6319	Genotoxicity	0.881
SR-MMP (%)	0.7–1.0	0–0.3	0.7–1.0	ADMETlab 3.0	5913	Oxidative stress	0.933
SR-p53 (%)	0.7–1.0	0–0.3	0.3–0.7	ADMETlab 3.0	6915	Tissue integrity	0.898
hERG I inhibitor	No	No	No	pkCSM 1.0	368	Cardiac toxicity	0.881
Herg II inhibitor	Yes	Yes	Yes	pkCSM 1.0	806	Cardiac toxicity	0.876
Oral rat chronic toxicity (log)	1.239	0.932	1.303	pkCSM 1.0	445	Long exposure toxicity	n/a
MTD (log)	0.624	0.292	0.483	pkCSM 1.0	1220	Maximum tolerated dose/day	n/a
T. pyriformis toxicity (log)	0.287	0.285	0.285	pkCSM 1.0	1571	Lethal concentration (LC_50_)	n/a
Minnow toxicity (log)	0.577	−0.417	1.995	pkCSM 1.0	554	Lethal concentration (LC_50_)	n/a
Cardiotoxicity (binary)	Nonblocker	Nonblocker	Blocker	PRED-hERG 5.0	14 831	Cardiac toxicity	0.970
Cardiotoxicity (multiclass)	Moderate blocker	Weak blocker	Moderate blocker	PRED-hERG 5.0	14 831	Cardiac toxicity	0.970
Cardiac failure	0.000	0.000	0.333	Adverpred 1.0	904	Cardiac toxicity	0.856

## Results and discussion

**Figure 1.**
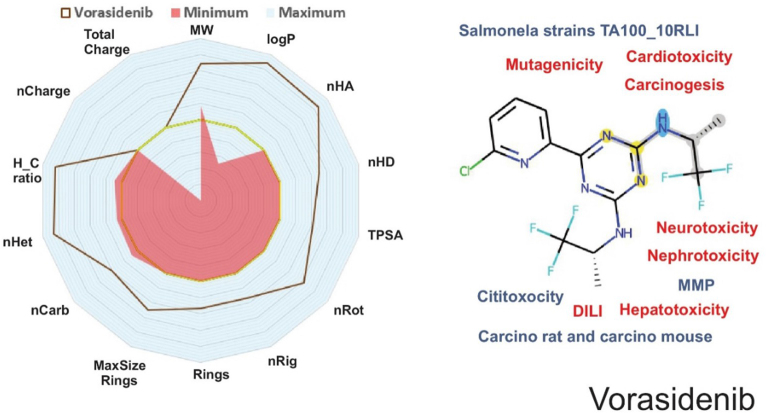
Physicochemical and ADMET descriptors of Vorasidenib utilizing data from webservers and databanks. A radar plot depicting some of the main physicochemical properties is presented on the left. On the right, the molecule’s 2D structure with some of its main ADMET attributes, with blue denoting properties that are within reference values, and red denoting potential toxicity.

Understanding a compound’s physicochemical and pharmacokinetic features is essential for analyzing its drug-likeness properties.^[^6-9^]^ Vorasidenib exhibited a molecular weight (MW) of 414.08 g/mol, a logP of 5.29, and numbers of hydrogen bond acceptors (nHA) and donors (nHD) of 6 and 2, respectively. According to Lipinski’s Rule of Five, a drug should have a MW ≤ 500, a logP ≤ 5, nHA ≤ 10, and nHD ≤ 5 (Fig. 1), with up to one violation considered acceptable. Vorasidenib violates one of these criteria (logP > 5) but is still considered to have acceptable drug-likeness. However, according to Hughes et al (2008) and Yukawa and Naven (2020), compounds with a low topological polar surface area (TPSA < 75) combined with a high logP (>3) can have toxic effects up to 2.5 times higher than those of drugs within safer ranges^[^37-39^]^. Vorasidenib has a logP of 5.29 and a TPSA of 75.62, indicating a potential for increased toxic effects.

Our ADMET analysis suggests that Vorasidenib may pose a risk for drug-induced liver injury (DILI) and hepatotoxicity. The liver is crucial for drug metabolism and detoxification, and compounds with certain structural or physicochemical properties may lead to hepatocellular damage. *In silico* predictions indicate that Vorasidenib possesses properties associated with hepatic risk factors that may lead to liver damage during and after treatment. This is particularly important as DILI accounts for up to 50% of acute liver failure cases^[^40^]^. Understanding the impact of Vorasidenib on the liver is crucial for patients undergoing long-term use, especially if they require combination therapy, increasing the risk of developing drug-induced fatty liver disease (DIFLD)^[^41^]^.

Corroborating our toxicity predictions, the most significant clinical study with Vorasidenib, the phase III INDIGO trial, indicates its potential to cause hepatic transaminase elevations, which can lead to hepatic failure, hepatic necrosis, and autoimmune hepatitis^[^24^]^. In the pooled safety population, 58% of patients treated with Vorasidenib experienced increased alanine aminotransferase (ALT) levels, and 44% experienced increased aspartate aminotransferase (AST) levels. Grade 3 or 4 increased ALT or AST occurred in 9% and 4.8% of patients, respectively. Among these patients, 4.1% (10/244) had concurrent Grade 3 to 4 ALT or AST elevations. Additionally, 34% of patients had increased gamma-glutamyl transferase (GGT), with 2.2% experiencing Grade 3 or 4 elevations. Bilirubin increases occurred in 4.8% of patients, with 0.4% at Grade 3 or 4 levels. Nine percent of patients had an increase in alkaline phosphatase, with 0.9% at Grade 3 or 4 levels.

Two patients met the laboratory criteria for Hy’s Law, with concurrent elevations in ALT or AST more than three times the upper limit of normal (ULN) and total bilirubin more than two times the ULN, events associated with autoimmune hepatitis and hepatic failure. The median time to the first onset of increased ALT or AST was 57 days (range: 1–1049).

Permanent discontinuation of Vorasidenib was required for 2.9% of patients due to ALT elevations, 1.6% for AST elevations, and 0.4% for GGT elevations. Dosage reductions were necessary in 7% of patients with ALT elevations, 1.2% with AST elevations, and 0.4% with GGT elevations. Dosage interruptions were required in 14% of patients with ALT elevations, 6% with AST elevations, and 1.6% with GGT elevations.

Similarly, in the phase I trial by Mellinghoff et al (2021), dose-limiting toxicities (DLTs) were predominantly associated with elevated transaminases at doses ≥100 mg^[^21^]^. These hepatic events were dose-dependent and reversible upon dose modification or discontinuation, validating our *in silico* predictions regarding potential hepatotoxicity.

Based on the predicted hepatotoxicity profile and FDA-Approved Drugs informations (Drugs@FDA—https://www.accessdata.fda.gov/scripts/cder/daf/index.cfm. Labels for NDA 218 784. Present in: https://www.accessdata.fda.gov/drugsatfda_docs/label/2024/218784s000lbl.pdf. Access: November 24, 2024), we recommend practical monitoring strategies to mitigate risks:

1. **Liver function tests (LFTs**): Monitor ALT, AST, GGT, total bilirubin, and alkaline phosphatase levels prior to initiating Vorasidenib treatment, every 2 weeks during the first 2 months, then monthly for the first 2 years of treatment, and as clinically indicated. More frequent testing should be performed in patients who develop transaminase elevations.
2. **Thresholds for dose modification**:
  - If ALT or AST levels are ≥3 × ULN: Increase monitoring frequency; consider reducing the dose of Vorasidenib.
  - If ALT or AST levels are ≥5 × ULN: Temporarily withhold Vorasidenib; Resume treatment at a lower dose once liver enzymes have returned to baseline levels.
  - If ALT or AST levels are ≥20 × ULN or in cases of significant bilirubin elevation: Permanently discontinue Vorasidenib; Evaluate the patient for potential liver injury.

In addition, our predictions indicate that Vorasidenib has low excretion rates associated with a low maximum recommended dose, which may lead to accumulation in the bloodstream over time. Renal excretion of unchanged drugs is an important route for drug elimination. Ideal renal clearance (CLr) values exceed 5.0 ml/min/kg to efficiently excrete drugs and reduce prolonged exposure^[^42,43^]^. Drugs with CLr values below this threshold remain in the body for extended periods, potentially increasing the likelihood of toxic reactions. This emphasizes the importance of dose adjustments and careful therapeutic monitoring to mitigate risks associated with drug accumulation^[^44^]^.

Vorasidenib may also have effects on neurotoxicity, nephrotoxicity, and cardiotoxicity. Since the drug targets neoplastic cells in the brain, its potential neurotoxic effects are of concern because interaction with neuronal tissue may inadvertently cause neuronal damage or impair normal neurological function^[^45^]^. In the phase I trial, neurotoxic events such as seizures were reported in 28.8% of patients with glioma treated with Vorasidenib, with 7.7% experiencing grade ≥3 seizures^[^21^]^. This supports our in silico predictions of potential neurotoxicity.

Although Vorasidenib’s improved CNS penetration and relatively favorable toxicity profile position it as a compelling alternative to Ivosidenib and Enasidenib, its previously mentioned higher likelihood of carcinogenicity (0.634) raises critical concerns for long-term use, and measures should be implemented during long-term treatments to avoid further health complications in patients. During clinical practice, this risk may be mitigated by more frequent imaging studies – such as MRI or computed tomography scans—, aiming for an early detection of secondary malignancies, as well as the dosage of regular biomarker (e.g. circulating tumor DNA and other relevant oncogenic markers). In addition, our results suggest that nephrotoxicity and neurotoxicity could also be a cause of concern that may require additional measures and the implementing of more specific monitoring strategies. Performing regular serum creatinine and eGFR assessments over the course of the treatment may help detect subtle renal impairment before it progresses, while electroencephalographic monitoring and cognitive assessments could help detect the early onset of neurological side effects.

Regarding cardiotoxicity, our analysis suggests that Vorasidenib may interact with the human Ether-à-go-go-Related Gene (hERG) channels and is classified as a hERG II blocker, which is associated with risks such as QT interval prolongation and arrhythmias^[^46^]^. However, in the clinical trials conducted by Mellinghoff et al (2021) and DiNardo et al (2023), significant cardiotoxic events were not reported^[^21,47^]^. This discrepancy indicates that while in silico models predict a potential risk, it has not been observed clinically to date.

Nephrotoxicity was also not significantly reported in clinical studies, suggesting that the predicted nephrotoxic risk may be lower than anticipated or may require longer-term studies to manifest.

Building upon our findings that in silico predictions of hepatotoxicity align with clinical data, it is imperative to extend our focus to the theoretical nephrotoxicity of Vorasidenib. Although Vorasidenib has been approved by the FDA, its pharmacokinetics and safety profile in patients with creatinine clearance (CLcr) ≤ 40 mL/min or those requiring dialysis have not been thoroughly investigated (). For these patient populations, increased vigilance in monitoring adverse reactions is essential, and dosage modifications should be considered as necessary to mitigate potential nephrotoxic risks. The FDA currently recommends no dosage adjustment for patients with CLcr >40 mL/min; however, our analysis suggests that patients below this threshold may benefit from tailored dosing strategies to enhance safety.

Furthermore, comprehensive safety evaluations encompassing long-term renal function assessments and pharmacogenomic studies are crucial for understanding Vorasidenib’s full toxicological profile. Integrating Vorasidenib into clinical protocols should also involve exploring combination therapies that may offer synergistic benefits while minimizing adverse effects. Additionally, refining patient selection criteria based on genetic and renal function markers will ensure that Vorasidenib is administered safely and effectively, maximizing therapeutic outcomes and minimizing risks.

A study by Craveiro et al (2019) associated multiple levels of toxicity with drugs that were withdrawn from the market, with a significant proportion linked to high degrees of cardiotoxicity, nephrotoxicity, and neurotoxicity^[^48^]^. This raises a potential concern for Vorasidenib, underscoring the importance of comprehensive clinical evaluations and vigilant patient monitoring. Ensuring the early detection and management of any adverse events is essential to prevent organ function impairment during treatment and to maintain the drug’s accessibility to the public.

Concerns regarding Vorasidenib include its potential for genotoxicity and carcinogenicity. *In silico* analyses suggest that Vorasidenib may induce DNA damage or mutations, potentially contributing to cancer development, particularly in patients already predisposed to tumor formation^[^49^]^. Assessing these risks during early-phase clinical trials is challenging due to their short duration and limited patient populations. Thus, long-term studies and postmarketing surveillance are crucial to fully evaluate these potential adverse effects.

The withdrawal of medications such as troglitazone, a type 2 diabetes treatment removed due to hepatotoxicity undetected in initial trials, underscores the importance of robust safety monitoring post-approval. Similarly, new adverse effects continue to emerge for recently approved drugs, reinforcing the need for vigilant pharmacovigilance^[^50-52^]^. For Vorasidenib, thorough genotoxicity and carcinogenicity testing is essential to ensure its therapeutic benefits outweigh potential risks, especially given the FDA’s precedent for withdrawing drugs confirmed to have carcinogenic properties^[^53^]^.

Our study underscores the importance of integrating computational toxicology with clinical safety data to provide a holistic risk assessment for Vorasidenib. The concordance between our *in silico* predictions and observed clinical adverse events, particularly hepatotoxicity and neurotoxicity, reinforces the validity of computational approaches. However, discrepancies in predicted and observed cardiotoxicity and nephrotoxicity highlight the need for continuous monitoring and validation of *in silico* models with real-world clinical data. As Vorasidenib moves into broader clinical use following FDA approval, postmarketing surveillance will be indispensable in confirming its long-term safety profile and addressing potential risks predicted by *in silico* methods.

To validate our *in silico* results and highlight the potential of Vorasidenib as a novel therapeutic agent, we compared its toxicity profile with that of other available, namely Ivosidenib and Enasidenib, whose toxicity profiles are already well reported in the literature. This comparison underscores the prospects of Vorasidenib as a potential alternative for the treatment of IDH-mutated gliomas (Table 1).

Vorasidenib showcases a more favorable cardiotoxicity profile compared to its counterparts. Ivosidenib, although also a nonblocker (53.32%), has a higher probability of hERG blockade (0.875), while Enasidenib is classified as a blocker (54.08%) with a probability of 0.516. Both Vorasidenib and Ivosidenib have a probability of heart failure of 0, while Enasidenib has a probability of 0.333, indicating a potential risk. These results suggest that Vorasidenib may have a lower risk of cardiotoxic effects when compared to the other two drugs.

In terms of hepatotoxicity and DILI, Vorasidenib has lower probabilities than both Ivosidenib (hepatotoxicity 0.964, DILI 0.798) and Enasidenib (hepatotoxicity 0.973, DILI 0.515). Although hepatotoxicity is predicted for all three drugs, the lower probabilities of Vorasidenib indicate a lower risk of liver toxicity, which is particularly important for long-term therapy and patient safety, especially those already undergoing surgical procedures. These findings are on par with known hepatotoxic risks associated with Ivosidenib and Enasidenib, with the latter being related to cases of hyperbilirubinaemia^[^18^]^.

Vorasidenib also has a higher MTD of 0.624 log mg/kg/day, suggesting a wider safety margin compared to Ivosidenib (0.292) and Enasidenib (0.483), allowing for safer and more flexible dosages. Regarding nephrotoxicity, Vorasidenib has a probability of 0.963, slightly lower than Ivosidenib (0.990) and Enasidenib (0.997). For drug-related neurotoxicity, Vorasidenib has a probability of 0.894, which is lower than Ivosidenib (0.918) but higher than Enasidenib (0.75). Although all values indicate a significant risk, Vorasidenib performs relatively well compared to current therapies.

Despite these positive aspects, Vorasidenib has higher probabilities for certain toxic parameters. It has noteworthy increased probability of carcinogenicity of 0.634, exceeding that of Ivosidenib (0.244) and Enasidenib (0.294), indicating a greater potential risk that warrants further evaluation, especially for the treatment of patients already suspectible to oncogenesis. In terms of respiratory toxicity, Vorasidenib has a probability of 0.795, higher than Ivosidenib (0.3) but lower than Enasidenib (0.997), on par with clinical results indicating that Ivosidenib and Enasidenib may be associated with cases of differatiaton syndrome^[^18,54^]^. These results indicate areas where Vorasidenib may have disadvantages compared to the other agents, emphasizing the need for continued evaluation and careful monitoring.

In summary, the comparative analysis suggests that Vorasidenib has a more favorable toxicity profile compared to Ivosidenib and Enasidenib in key areas, including cardiotoxicity, hepatotoxicity, and maximum tolerated dose. These advantages, combined with its efficacy in the penetration of the central nervous system, make Vorasidenib a promising alternative for the treatment of IDH-mutated gliomas. However, the higher likelihood of carcinogenicity and probable nephrotoxicity profiles raises concerns that may require further investigation.

Although computational approaches may provide valuable preliminary insights into drug-likeness and toxicity, several limitations inherently associated with *in silico* methods must be recognized. First, *in silico* predictions rely heavily on existing databases and known structure–activity interactions that may not fully represent the complexity of biological systems or novel chemical structures, and may not correctly represent those interactions in *in vivo* or *in vitro* situations. This is particularly true for Vorasidenib, where some predicted toxicities (e.g. hERG inhibition and nephrotoxicity) did not match clinical trial observations, evidencing the gap between computational findings and real-world outcomes. Additionaly, current computational models may not adequately account for patient-specific factors such as genetic polymorphisms involving drug-degrading enzymes (e.g., CYP2C19, CYP3A4) or transport proteins (e.g., P-gp), comorbidities or drug–drug interactions – especially patients undergoing polypharmacy treatments – which can significantly influence toxical properties. For instance, while our analysis predicted potential nephrotoxicity, no significant adverse renal events were reported in clinical trials, suggesting potential limitations with the predicted models. Lastly, the absence of experimental validation for many predicted parameters, especially for long-term effects such as carcinogenicity and chronic organ toxicity, requires cautious interpretation. Cofounding factors such as the influence of metabolites, tissue-specific distributions and complex biological pathways may not be fully represented in current simulated models. This is exemplified by some discrepancies observed between the predicted mechanisms of nephrotoxicity and the actual clinical manifestations observed in the INDIGO trial^[^21,47^]^. Finally, the dynamic nature of drug–target organ interactions and the potential for adaptive biological responses cannot be fully captured by static computational models, highlighting the need for integrated approaches that combine *in silico* predictions with experimental and clinical validation. Continued research backed up with experimental data are essential to fully characterize the safety profile of Vorasidenib and ensure its optimal integration into clinical practice.

## Conclusion

Our comparative analysis provides valuable insights into the pharmacokinetic and ADMET properties of Vorasidenib, and its comparison with other currently available drugs. The results suggest that while Vorasidenib has favorable properties in terms of oral bioavailability and permeability, certain aspects such as low water solubility and potential off-target interactions as well as adverse toxicity properties such as nephro-, neuro- and cardiotoxicity and carcinogenesis risks require increased attention. As such, its dual inhibitory effect on IDH1/2, good CNS penetration as shown in our results, and lower toxicity endpoints if compared with other antineoplastic drugs currently available as showcased in clinical trials and our analysis could reshape current treatment by offering a more targeted approach with fewer adverse effects. We recommend conducting further clinical studies to validate these predictions and optimize dosing strategies. Careful monitoring of patients for adverse effects is essential to ensure the safe and effective use of Vorasidenib in the treatment of IDH1/2-mutated gliomas.

## Research registration unique identifying number (UIN)

1. Name of the registry: Not required
2. Unique Identifying number or registration ID: Not required
3. Hyperlink to your specific registration (must be publicly accessible and will be checked): Not required
